# Purification and characterization of novel fibrinolytic proteases as potential antithrombotic agents from earthworm *Perionyx excavatus*

**DOI:** 10.1186/2191-0855-1-26

**Published:** 2011-09-30

**Authors:** Tram Thi Bich Phan, Tien Duy Ta, Dung Thi Xuan Nguyen, Lambertus AM Van Den Broek, Giang Thi Huong Duong

**Affiliations:** 1 College of Agriculture and Applied Biology, Can Tho University, Can Tho, Vietnam; 2 Faculty of Food Processing Technology, Can Tho University of Technology, Can Tho, Vietnam; 3 Biotechnology Research and Development Institute, Can Tho University, Can Tho, Vietnam; 4 Wageningen UR Food & Bio-based Research, 6708 WG, Wageningen, The Netherlands

**Keywords:** chromatography, fibrinolysis, *Perionyx excavatus*, PMSF, serine protease, tandem MS analysis

## Abstract

Six protease fractions, namely FI, FII, FIII-1, FIII-2, FIII-3 and FIV, were isolated from *Perionyx excavatus *earthworm biomass by acetone precipitation, followed by serial chromatography using anion exchange, hydrophobic interaction and size exclusion chromatography. All fractions exhibited strong hydrolytic activity towards casein. The activity of six fractions towards fibrin, determined by fibrin plate assay, ranged from 44 to 831 plasmin unit.mg^-1 ^and ranked as FIII-3 > FIII-2 > FI > FIII-1 > FIV > FII. Casein degradation was optimal at pH 7 and 11, and at 45-60°C. All fractions were considerably stable at high temperature and wide pH range. They were completely inhibited by phenylmethylsulfonyl fluoride (PMSF). The molecular weights (MW) and isoelectric points (pI) determined by 2D-electrophoresis were 27.5-34.5 kDa, and 4.3-5.2, respectively. Tandem mass spectrometry (MS) analysis was used to deduce the amino acid sequences of some peptides from FIII-1 and FIII-2. The sequences shared 16.9% and 13.2% similarity, respectively, with the fibrinolytic enzymes from two related earthworm species, *Lumbricus rubellus *and *Eisenia fetida*. The *P. excavatus *proteases were classified as serine proteases. They could perform rapid hydrolysis on both coagulated fibrous fibrin and soluble fibrinogen monomers without the presence of activators such as tPA or urokinase.

## Introduction

Cardiovascular diseases have become one of the biggest concerns all over the world (Grundy et al. 1999). Among these, thrombosis is the most widespread within the elderly population. The disease results from severe blood-clotting, which leads to obstruction of the blood flow circulation. In the physiological state, fibrin and platelets are utilized for clotting to prevent blood loss from injuries in a process called hemostasis (Furie and Furie 2008). A serine protease called plasmin acts to digest blood clots via fibrinolysis to properly terminate the hemostasis. Plasmin deficiency is one reason that leads to thrombosis due to insufficient clots degradation.

Fibrin is a fibrous polymer protein that plays an important role in the final blood coagulation step in hemostasis. The fibrinogen monomer is a 304 kDa glycoprotein containing two sets of three different chains: Aα, Bβ and γ (Mosesson 2005). The conversion of fibrinogen into fibrin requires the presence of thrombin, a serine protease that cleaves the N-terminus of Aα and Bβ chain (Mosesson et al. 2001). Fibrinogen level was reported to be significantly related to the incidence of cardiovascular disease in both men and women during the tenth biennial examination of the Framingham Study (Kannel et al. 1987). Treatment of cerebral venous thrombosis currently relies on the use of anticoagulants such as heparin, which is also a medicament for deep vein thrombosis (Stam et al. 2003) despite the risk of consequent occurrence of intracranial hemorrhage (Einhaupl et al. 1991 and Mehraein et al. 2003). Enzyme therapy of thrombosis has been investigated since 1969 by using streptokinase, a fibrinolytic enzyme (Kakkar et al. 1969), and was reported to be a better treatment for acute deep vein thrombosis than of heparin (Marder et al. 1977 and Arnesen et al. 1982).

A novel fibrinolytic enzyme, namely lumbrokinase, has been isolated from some earthworm species such as *Lumbricus rubellus *(Cho et al. 2004; Mihara et al. 1991 and Nakajima et al. 1983) and *Eisenia fetida *(Yang and Ru 1997), and was thoroughly characterized. They have been identified as serine protease isozymes, which are highly thermostable and alkali tolerant. The genes encoding strong fibrinolytic enzymes from these earthworms have been identified (Dong et al. 2004). High-throughput production of these enzymes by recombinant DNA technology has been conducted in *Escherichia coli *(Cho et al. 2004 and Xu et al. 2010) and *Pichia pastoris *(Ge et al. 2005 and Sugimoto et al. 2005). The recombinant enzymes expressed strong fibrinolytic activity both in vitro (Sugimoto et al. 2005) and in vivo in rats via oral administration (Cho et al. 2004). Crystallographic data of two components of *L. rubellus *lumbrokinase were obtained, revealing the structure determinants of their catalytic mechanisms (Tang et al. 2002 and Wang et al. 2005). The analysis showed that the structure of component B resembled that of the trypsyin-like proteases, and was the first reported glycosylated trypsin-like structure. The study also revealed the structural basis for high stability and complicated post-translational modifications of the enzyme.

Application of the fibrinolytic enzymes has also been of great interest in Vietnam, focusing especially on the use of local enzyme sources. The preliminary experiments of this research have discovered the novel and remarkably strong firbinolytic enzymes from *Perionyx excavatus *earthworm (family Megascolecidae). This species has been widely cultivated in Southern Vietnam for the production of aquatic animal feeds and compost. In our study, an extensive purification of these proteases was carried out together with the characterization of temperature and pH optimum, inhibition, substrate specificity, fibrinolytic effect and partial sequencing. We have found the potential of these enzymes as effective fibrinolytic agents. The study was thus aimed to understand more deeply the properties of these enzymes and to initially evaluate their applicability in thrombosis treatment.

## Materials and methods

### Materials

Living earthworms (*P. excavates*; 8-9 weeks old) were purchased from the farms in An Giang province, Vietnam. Protease substrates included casein from Merck (USA), fibrinogen (together with thrombin and human plasmin), *N*α -benzoyl-L-arginine *p*-nitroanilide (BA*p*NA), and *N*α -benzoyl-L-tyrosine *p*-nitroanilide (BT*p*NA) from Sigma-Aldrich (USA). The enzyme inhibitors were bought from Sigma (USA). The 2D-electrophoresis kits were obtained from Bio-Rad (USA).

### Autolysis of *P. excavatus*

Earthworm biomass was washed and finely homogenized in distilled water containing 0.1% (w/v) sodium azide. Autolysis was initially performed at 45°C for 4 hours and continued at room temperature for 15 days with stirring. Enzyme activity was measured daily by the modified method of Anson (Anson 1938) using casein as substrate. One activity unit (U) was defined as the amount of enzyme that catalyzed the release of 1 μmol of tyrosine per minute at 30°C at pH 7.5. Specific activity (expressed in U.mg^-1^) was defined as the activity per milligram of total protein, which was determined by the Lowry protein assay (Lowry 1951).

### Purification of *P. excavatus *proteases

The autolysate was centrifuged (11,200 × g) at 4°C for 30 minutes, and the supernatant was precipitated in pre-chilled acetone at 4°C for 2 hours. The precipitate was collected by centrifugation (11,200 × g) at 4°C for 30 minutes and was lyophilized. A series of chromatographic techniques were used for purification. Fractions containing proteases were collected and analyzed by SDS-PAGE (Hames 1998) after each purification step.

#### Anion exchange chromatography (AEX)

Precipitated protein was re-dissolved in 20 mM Tris-HCl buffer pH 8.5 and was loaded on to a 1.5 × 40 cm Unosphere Q (Bio-Rad, USA) column at a flow-rate of 0.8 ml.min^-1^. Bound proteins were eluted in the same buffer, with a continuous NaCl gradient from 0 to 0.45 M at a flow-rate of 1 ml.min^-1^.

#### Hydrophobic interaction chromatography (HIC)

Each active fraction was dialyzed against 20 mM Tris-HCl buffer pH 8.5 for desalting; subsequently 30% (w/v) ammonium sulfate (AS) was added, followed by loading the sample on to a 1.5 × 30 cm Phenyl Sepharose (GE Healthcare, UK) column at a flow-rate of 1 ml.min^-1^. Elution was done using a continuous AS gradient from 30% to 0% at the same flow-rate.

#### Size exclusion chromatography (SEC)

SEC was carried out for each active HIC fraction on a Superose 12 column (GE Healthcare, UK) using the Biologic HR System (Bio-Rad, USA). The running buffer was 20 mM Tris-HCl buffer pH 7.5 containing 15 mM NaCl, passing through the column at a flow-rate of 0.5 ml.min^-1^. Eluted fractions were dialyzed against water and freeze-dried using a VirTis Bench Top Manifold Freeze Dryer (SP Scientific, USA) and stored at -20°C for later use.

### Characterization of *P. excavatus *protease fractions

#### Determination of optimal temperature and thermostability

The caseinolytic assay was performed to determine the optimal temperature for each active SEC fraction in 50 mM sodium phosphate buffer pH 7.5 at different temperature ranging from 30°C to 80°C in 10 minutes. To investigate the thermal stability, each fraction was incubated at different temperatures between 37 and 70°C for 3 hours, and the remaining activity (expressed as % of the activity at 37°C) was determined after 30, 60, 120 and 180 minutes.

#### Determination of optimal pH and pH-tolerance

Each fraction was assayed for caseinolytic activity at 30°C in different pH-buffered solutions from 3 to 12, prepared as follows: 100 mM Glycine-HCl (pH 2), 100 mM citrate-phosphate (pH 3-5), 100 mM sodium phosphate (pH 6-8), 100 mM Glycine-NaOH (pH 9-11), and KCl-NaOH (pH 12-13). The pH tolerance was determined by incubating each fraction in these buffer solutions for 16 hours at 4°C; and after neutralization at 30°C the remaining activity was determined (expressed as % of the activity at pH 7). In addition, the long-term storage of these enzymes was also investigated using distilled water and 50 mM sodium phosphate buffer pH 7.5 as preservative solvents. The freeze-dried enzymes were dissolved in these solvents containing 0.1% (w/v) sodium azide, and stored in carefully-sealed glass vials at 4°C for 10 months. The remaining activity, displayed as % of the initial activity, was determined every month.

#### *Enzyme inhibition assay was *done with eight inhibitors

PMSF, *N*-torsyl-L-phenylalanine chloromethyl ketone (TPCK), aprotinin, leupeptin, soybean trypsin inhibitor (SBTI), ethylene diamine tetraacetic acid (EDTA), chymostatin and peptastatin. The used concentration ranged from 0.01 to 1 mM. Samples incubated with casein but without inhibitors were used as controls. Each fraction was incubated separately with each inhibitor in 50 mM sodium phosphate buffer pH 7.5 containing casein at 37°C for 10 minutes. The measured activity was compared with the controls.

#### Hydrolytic assay using different substrates

The hydrolytic ability towards different substrates such as casein, fibrin, BA*p*NA, and BT*p*NA was examined. The assays for the last two substrates were performed at 37°C in 50 mM sodium phosphate buffer pH 7.5 based on the method of (Wang et al. 2005). One activity unit (U) on these substrates was defined as the amount of enzyme that released of 1 μmol of *p*-nitroaniline under the given conditions. Fibrinolysis was performed on a fibrin plate as described by (Choi and Sa (2001)). Briefly, a mixture of 0.6% (w/v) fibrinogen and 2% (w/v) agar was prepared in 50 mM sodium phosphate buffer pH 7.5, boiled for 2 minutes, cooled down to 55°C and subsequently added with thrombin (10 NIH unit.ml^-1^) for coagulation in a Petri disc. Ten μl of enzyme solution was pipetted into the small holes created on the plate and incubated at 37°C for one hour. Fibrinolytic activity, expressed as plasmin unit (PlasminU), was extrapolated from the area of the hydrolytic zones based on a human plasmin standard curve. In vitro cleavage of fibrinogen was investigated by incubating each protease fraction (0.2 mg.ml^-1^) with bovine fibrinogen (1 mg.ml^-1^) at 37°C in 50 mM sodium phosphate buffer pH 7.5 containing 100 mM NaCl. Aliquots of 10 μl were taken out of the reaction mixture after 10, 20, 30, 60, 90, 120, 180, 240 and 480 minutes, and were visualized on SDS-PAGE gel.

#### 2D-electrophoresis-coupled MS/MS sequencing

The protease fractions were purified using a 2D-Cleanup Kit. Isoelectric focusing (IEF) dimension was run on pH 3-10 IPG-Strips, and the second dimension was carried out on a 12% polyacrylamide gel without stacking layer, according to the manufacturer's user manuals. Images were captured by GelDocTM XR+ system (Bio-Rad, USA) and were analyzed using PDQuestTM 2-D Analysis Software (BioRad, USA) for MW and pI calculation. Each protein spot was excised, digested with trypsin, and purified using the protocol from the Protein and Proteomics Centre (NUS, Singapore). Tandem MS analyses for these peptides were carried out at the Laboratory of Protein and Proteomics Centre (NUS, Singapore) using a MALDI-TOF-TOF MS: 4800 Proteomics Analyzer instrument (Applied Biosystems, Framingham, USA).

## Results

### Purification of *P. excavatus *proteases

The hydrolytic activity on casein of the crude extract before autolysis was determined as 0.02 U.mg^-1^. To examine the further activation of these proteases, earthworm biomass was subjected to autolysis at 45°C in 4 hours, following by a 15-day autolysis at room temperature. Consequently, a two-fold increase of caseinolytic activity was achieved after 10 days.

Precipitation of earthworm proteins from the autolysate by acetone was done after the completion of autolysis. The pre-chilled acetone:lysate 2:1 (v/v) ratio resulted in the highest recovery yield of proteins (31%). Although the yield was not very high, the total proteolytic activity was more or less the same as prior to precipitation (76.84 U in comparison to 81.05 U). The specific proteolytic activity was therefore increased three-fold (0.138 U.mg^-1 ^in comparison to 0.045 U.mg^-1^).

**Figure 1:**
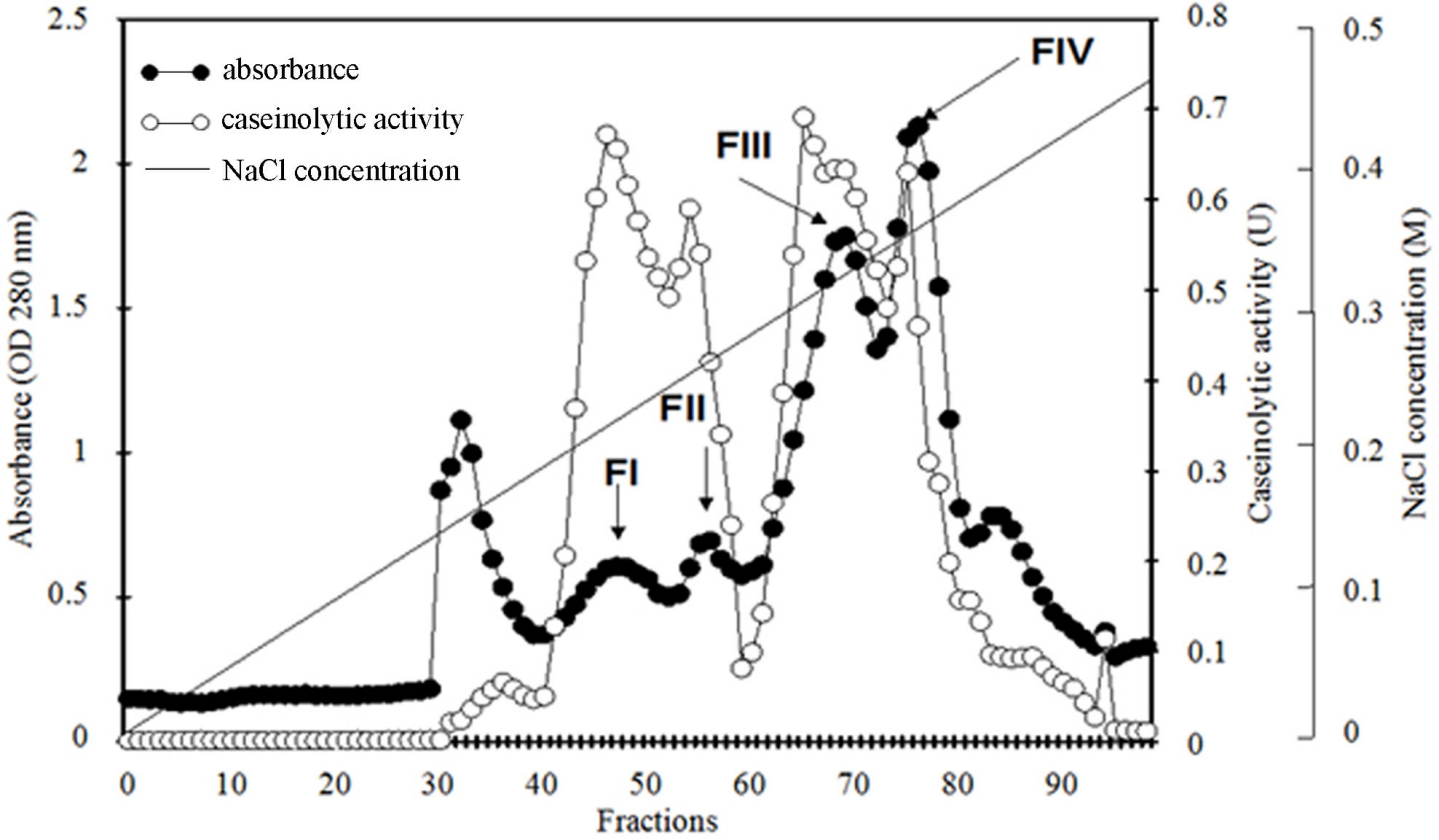
**Anion exchange chromatogram of *P. excavatus *crude proteins**. Four peaks indicated as FI, FII, FIII, and FIV (arrows) with caseinolytic activity were obtained when eluting with a continuous gradient of NaCl concentration (0-0.45 M).

After desiccation, earthworm proteins were dissolved in 20 mM Tris-HCl buffer pH 8.5 and subjected to AEX using a Unosphere Q column. Four fractions, namely from FI to FIV, possessing caseinolytic activity were collected when applying a NaCl gradient of 0-0.45 M (Figure 1). FI had the highest activity. SDS-PAGE profile of all fractions showed impurities (data not shown), thus requiring further purification processes.

**Figure 2:**
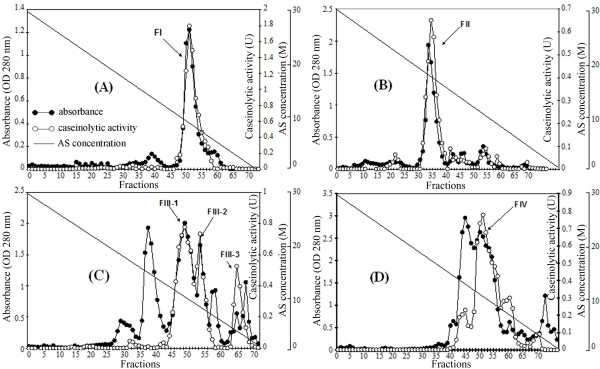
**Hydrophobic interaction chromatograms of four AEX fractions**. A continuous gradient of ammonium sulfate (AS) concentration (30-0%) was used for eluting. (A) FI and (B) FII were eluted as single prominent peaks. (C) FIII was separated into three sub fractions with protease activity: FIII-1, FIII-2 and FIII-3. (D) FIV was fractionated into multiple peaks, however, only one fraction showed protease activity. All active fractions were indicated by arrows.

Each fraction was loaded onto a phenyl sepharose column after dialysis, and a gradient of 30-0% AS concentration was used for elution. FI and FII were eluted in single prominent peaks (Figure 2A and 2B). In contrast, FIII was further separated into three fractions, namely FIII-1, FIII-2 and FIII-3, having caseinolytic activity (Figure 2C). FIV was fractioned into two partially overlapping peaks; however, only the later one had caseinolytic activity and thus was referred to as FIV (Figure 2D). After this step, FI and FII showed high purity as determined by SDS-PAGE (data not shown). The sub-fractions of FIII and FIV, however, still contained some low MW contaminants (data not shown). Thus a subsequent SEC was necessary to purify these fractions and to confirm the purity of the others as well.

**Figure 3:**
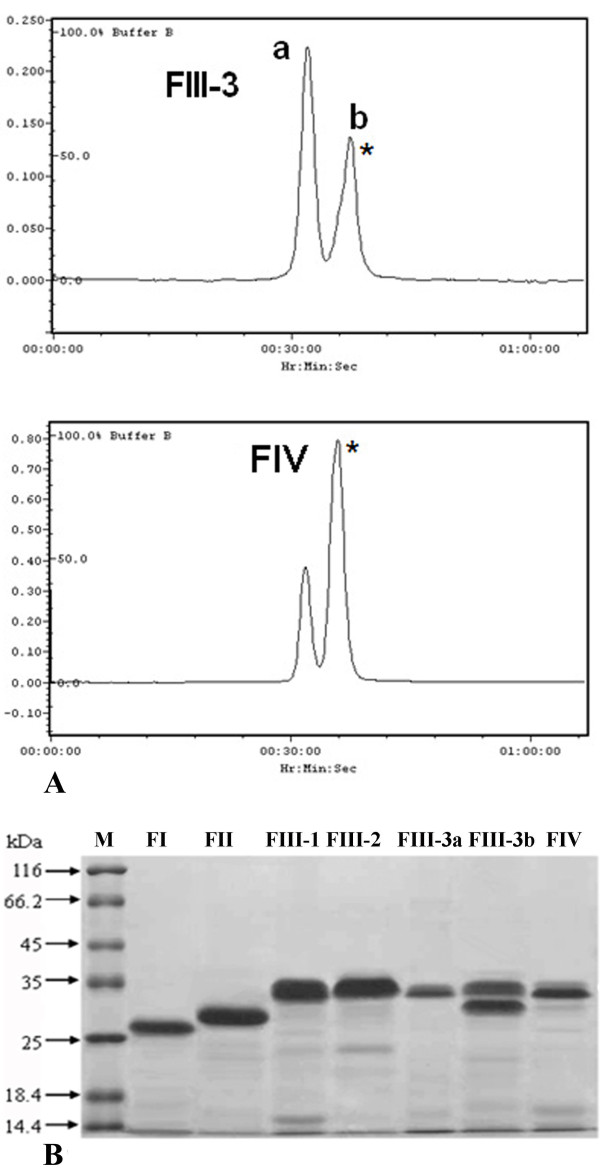
**Profiling of *P. excavatus *protease fractions**: (A) Size exclusion chromatogram of FIII-3 and FIV presented their fractionation into two peaks, ones of which marked with an asterisk showed protease activity. (B) SDS-PAGE profiling of all SEC fractions.

SEC was carried out for all active HIC fractions on a Superose 12 column. Three fractions FI, FII and FIII-1 eluted as a single peak with a MW of 28, 29 and 35 kDa, respectively. These results were in accordance with those obtained from SDS-PAGE (Figure 3B). The major peak of FIII-2 appeared to be a single band of 34 kDa on SDS-PAGE gel and showed protease activity. FIV was fractionated into two peaks but only the large peak of 34 kDa protein (marked as FIV*) showed proteolytic activity (Figure 3A). FIII-3 was also separated into two peaks, namely FIII-3a and FIII-3b, with a MW of 33 and 31 kDa, respectively, and only the latter one showed protease activity. Both fractions had a band of 33 kDa on SDS-PAGE gel. Besides, FIII-3b contained also a 31 kDa protein which was not present in FIII-3a, as seen in the SEC chromatogram. Most fractions were more than 98% pure as estimated by SDS-PAGE Coomassie Brilliant Blue staining. Only FIII-3b contained two proteins with more or less the same intensity. There were six proteolytic fractions in total that were purified. The same number of fractions was also reported for *L. rubellus *lumbrokinase using a similar purification strategy (Mihara et al. 1991).

### Effect of temperature and pH on the stability of *P. excavatus *proteases

**Figure 4:**
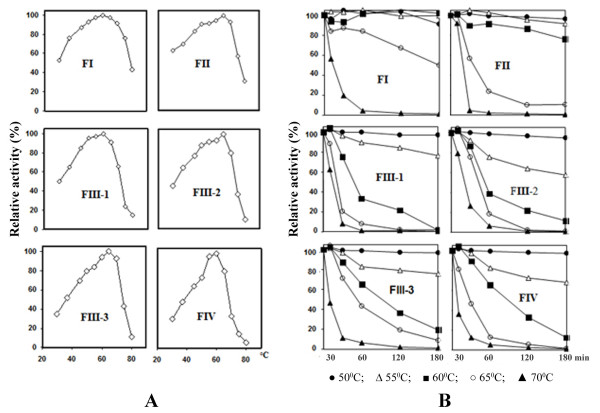
**Effect of temperature on the activity of *P. excavatus *proteases**: (A) Temperature optimum and (B) thermostability of all isolated fractions. The highest activity of each fraction in (A) and the activity of each fraction at 30°C in (B) were set at 100%.

All fractions exhibited maximal proteolytic activity in the temperature range of 60-65°C. Increasing the temperature to 70°C caused rapid loss of activity, and between 75-80°C almost complete inactivation was observed (Figure 4A). The thermostability over time was examined at different temperatures in a 3-hour period. These proteases were highly stable at temperature below 50°C but had rapid activity loss of 10-30% from 50°C onward. Higher temperatures inactivated these enzymes very rapidly. FI and FII were more stable than other fractions, as their activity decrease only about 10-20% at temperatures from 50 to 60°C (Figure 4B).

**Figure 5:**
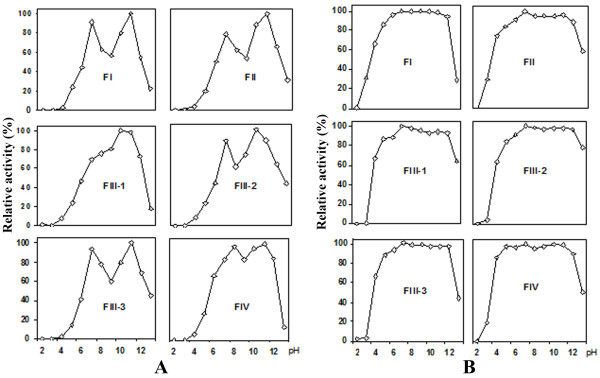
**Effect of pH on the activity of *P. excavatus *proteases**. All fractions except for FIII-1 had dual pH optima. The highest activity for each fraction at pH 7 was set at 100%, and the activities at other pH were calculated in accordance to this value.

All proteases expressed optimal activity at both pH 7 and 11, except for FIII-1 which showed the highest activity only at pH 7 (Figure 5A). The proteases of *P. excavatus *were stable in a wide pH range from 4 to 12 during 16 hours (Figure 5B). Similar results were reported for Korean and Japanese *L. rubellus *(Cho et al. 2004 and Mihara et al. 2004). FIII-1 and FIII-2 were the two most stable proteases towards a wide range of pH values in comparison to the others. The long-term preservation of the purified proteases at 4°C was experimentally investigated with two solvents, distilled water and sodium phosphate buffer pH 7.5. Sodium azide (0.1% w/v) was used as preservative. After 10 months, all fractions tended to be more stable in water (with only 10% activity loss) except for FIII-3, whose activity reduced to about 78% of the initial activity. In contrast, these enzymes were less stable in phosphate buffer since their activity decreased approximately 25-30% (data not shown).

### Inhibition of *P. excavatus *proteases

PMSF is known as an effective inhibitor for serine proteases. As shown in Table 1, this compound could almost completely inhibit all protease fractions, causing 92-100% of activity loss. In contrast, EDTA had no inhibitory effect on any of the six fractions, suggesting that they were not metalloproteases, because such enzymes need a metal ion for activity. Only FIII-3 was inhibited to some extent by TPCK, which is a specific inhibitor for chymotrypsin-like serine proteases. The other reversible inhibitors such as SBTI, aprotinin, leupeptin and chymostatin caused different levels of inhibition of these proteases.

**Table 1:** Effect of different inhibitors on *P. excavatus *proteases.

Inhibitors	Conc. (mM)	Relative activity (%)					
		
		FI	FII	FIII-1	FIII-2	FIII-3	FIV
Control		100	100	100	100	100	100
PMSF	1	0	0	2	4	0	8
TPCK	0.1	100	100	96	100	76	100
Aprotinin	0.01	29	95	87	95	26	71
Leupeptin	0.1	0	100	98	100	0	73
SBTI	0.01	0	76	81	93	0	0
EDTA	1	100	100	100	100	100	100
Chymostatin	0.1	19	100	76	100	19	90
Pepstatin	1	100	58	60	86	83	75

Relative activity was determined as percentage of the activity of enzyme without inhibitor (control) (results were rounded up to the integer)

### Hydrolytic activity on different substrates

All fractions were assayed for their hydrolytic ability towards different substrates including casein, fibrin, BA*p*NA, and BT*p*NA (Table 2). The last two are synthetic substrates specific for trypsin- and chymotrypsin-like proteases, respectively. None of these fractions showed hydrolytic activity towards BT*p*NA, indicating that they probably do not belong to the chymotrypsin-like protease group. This result was in accordance with the inhibitory assay (Table 1) although FIII-3 was to some extent inhibited by TCPK, a specific inhibitor for chymotrypsin-like serine proteases. Only three fractions FIII-1, FIII-2 and FIV were able to show hydrolysis of BA*p*NA.

**Table 2:** Hydrolytic activity of *P. excavatus *proteases on different substrates

	**Specific activity (U.mg**^-1^)			
**Fractions**	**Fibrin**	**Casein**	**BA*p*NA**	**BT*p*NA**

FI	602	1.8	0	0
FII	44	1	0	0
FIII-1	393	1	0.1	0
FIII-2	783	1	0.4	0
FIII-3	831	1.2	0	0
FIV	296	0.9	2.2	0

The caseinolytic activity was more or less the same for all fractions. Results from the fibrin plate assay, however, showed that all fractions had different levels of fibrinolytic activity. Three fractions FIII-3, FIII-2 and FI expressed the highest activity. Coagulated fibrin seemed not to be a specific substrate for FII since its hydrolysis towards fibrin was much smaller than those of the others.

**Figure 6:**
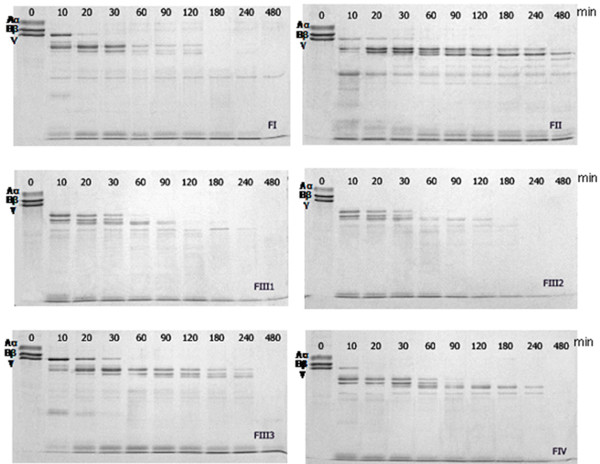
**Digestion of fibrinogen subunits (Aα, Bβ and γ) in time by *P. excavatus *proteases**. The MW of the three subunits Aα, Bβ and γ are 95, 56, and 51.5 kDa (UniProt), respectively. The time of hydrolysis is plotted at the top of the figures.

The hydrolytic effect of these proteases towards fibrinogen monomers was also investigated. As visualized on the SDS-PAGE gel, all fractions could completely degrade the Aα and Bβ subunits of fibrinogen within 10 minutes, and FIII-1 and FIII-2 could even cleave off the γ subunit. The hydrolytic ability of fraction FIII-2 was the highest since almost no protein bands were visible on the gel after 180 minutes (Figure 6). The hydrolytic activity was thus ranked as FIII-2 > FIII-1 > FI > FIV > FIII-3 > FII. The activity magnitude was more or less similar to the hydrolytic effect on intact fibrin in the fibrin-plate assay (Table 2).

### 2D-electrophoresis coupled MS/MS sequencing

Each protease fraction was analyzed on 2D-PAGE gel for determination of their MW and pI. All fractions, except for FI and FIII-3, appeared as single spots, indicating that they were pure. The pI values of these fractions were denoted in Table 3, ranging from 4.3 to 5.2. Interestingly, they shared similar pI's with the proteases from *E. fetida *(Zhao et al. 2006). The presence of two protein with similar MW but different pI (5.0 and 5.2), was observed for FI, thus it might contain two isozymes. Fraction FIII-3 was hardly visible on the gel, probably due to the loss during sample preparation for 2D-electrophoresis. Therefore, the pI and MW of this fraction could not be determined.

**Table 3:** The values of pI and molecular weights (MW) of all *P.excavatus *proteases, except for FIII-3, determined by PDQuest™ 2-D Analysis Software (Bio-Rad, USA).

Fractions	pI	MW (kDa)
FI (spot 1)	5.0	27.5
FI (spot 2)	5.2	27.5
FII	4.3	29.0
FIII-1	4.5	34.5
FIII-2	4.3	33.5
FIII-3	not determined	
FIV	4.5	34.0

**Figure 7:**
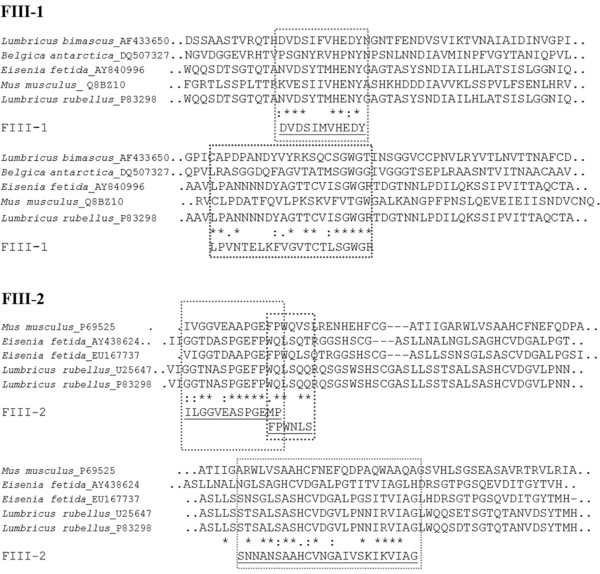
**Sequence alignment of fragments obtained from MS/MS analyses of FIII-1 and FIII-2 from *P. excavatus *with lumbrokinase and its precursor from *L. rubellus *(sp:P83298 and U25647) and *E. fetida *(gpu:EU167737 and AY438624), with a serine protease from *M. musculus *(sp:P69525 and Q8BZ10) and *B. antarctica *(gpu:DQ507327)**. Symbols were defined as following: (*) for identical amino acids, (.) for redundant amino acids with similar 3D-structures, (:) for redundant amino acids with similar physicochemical properties. Significantly similar sequences were wrapped in dot-lined boxes.

Only the MS/MS spectra of FIII-1 and FIII-2 peptide fragments were obtained with good signal-to-noise ratios. The sequence alignments revealed that they shared considerable similarity (16.9% and 13.2%, respectively) with the segments of fibrinolytic lumbrokinase isozyme C (EC 3.4.21) from *L. rubellus *and *E. fetida *(Figure 7). This enzyme is a serine protease with the length of 242 amino acid residues and a MW of 26 kDa. The two fractions FIII-1 and FIII-2 also showed sequence similarity, but at a lower degree, with the serine proteases from mouse (*Mus musculus*). The spectra obtained from the other fractions gave no clear signals, so it was not possible to determine any of these peptide sequences.

## Discussion

### Initial autolysis is necessary for full activation of *P. excavatus *proteases

As we could see, the proteolytic activity towards casein increased and peaked through a 15-day autolysis. The presence of sodium azide could inhibit the bacterial growth. The autolytic process could therefore trigger the release of proteases from the earthworm's tissues and exert a subsequent degradation of keratin and lipids, thus reducing the mixture's viscosity. The increase of proteolytic activity over time suggested that activation of these enzymes had occurred, probably through self-proteolysis that cleaved parts of the zymogens.

### Purification protocol for *P. excavatus *proteases

The acetone precipitation of the *P. excavatus *lysate was more effective than ammonium sulfate (AS) precipitation because of more impurity removal, higher proteolytic activity recovery (data not shown) and less time consuming since subsequent dialysis is not necessary.

The presence of the 33 kDa protein in faction FIII-3 could support the hypothesis of zymogen degradation mentioned earlier since it could be the zymogen of the 31 kDa peptide, and its presence in the SEC fraction probably resulted from the incomplete initial autolysis. Similar results were reported in the study on *L. rubellus *earthworm (Cho et al. 2004) in which a 44 residues were cleaved off from a 283-residue-zymogen to release the fully active proteolytic enzyme. This hypothesis, however, requires further validation through sequencing of our FIII-3a and FIII-3b proteins.

Generally, the two-step chromatography of AEX and HIC was sufficient for the purification of FIII-1, FIII-2, and FII, since their SEC and SDS-PAGE profiles represented pure proteins. Fraction FI actually contained two isozymes with close MW and pI, which could not be further separated. For FIII-3 and FIV, SEC was necessary to achieve the highest purity.

### *P. excavatus *proteases possess dual pH optima

The dual pH optima has not been reported for the proteases from *L. rubellus *and *E. fetida*. However, this characteristic was found for the intestinal serine protease of red flour beetle (*Tribolium castaneum*), whose optimal pH was determined to be at 4 and 8.5 (Oppert et al. 2003). (Choi et al. (1989)) studied the proteases from the parasitic protozoa *Toxoplasma gondii *and discovered that they catalyzed most effectively at pH 6 and 8.5. Likewise, the dual pH optima characteristic has been observed in various hydrolases such as β-glucuronidase from human seminal plasma (Gupta and Singh 1983), *Staphylococcus *sp. xylanase (Gupta et al. 2000), reptile lysozyme (Thammasirirak et al. 2006) and *Rhizopus *lipase (Upadhyay et al. 1989).

(Gupta et al. (2000)) hypothesized that the xylanase in their study might contain two distinct active sites that could perform catalysis at two distinct pH levels of 7.5 and 9.2, respectively, although no such enzyme has been reported before. In another study, the aspartate protease Plasmepsin I from *Plasmodium falciparum *was characterized, revealing the existence of two states of this protease as monomer and aggregated oligomer (Xiao et al. 2007). These two co-existing states resulted in the dual pH optima of the enzyme as determined experimentally. Since all protease fractions from *P. excavatus *in our study were completely inhibited by PMSF (Table 1), they would not harbor any active sites rather than the typical catalytic triad of serine proteases. Additionally, no aggregation was observed by SEC chromatography performed at pH 8.5. We therefore hypothesize that the *P. excavatus *proteases existed in both monomeric and aggregated oligomeric form in our assays; and the aggregation might be triggered at strong alkaline pH, for instance pH 11.

### Serine proteases

The inhibitory effect of PMSF towards all fractions revealed that *P. excavatus *proteases are serine proteases, since PMSF is a specific irreversible inhibitor for this group of proteases (James 1978). FIII-3 was to some degree inhibited by TPCK, which is specific for chymotrysin-like protease (Table 1). However, it was not able to hydrolyze the chymotrypsin-like specific BT*p*NA substrate. Therefore, it was not possible to classify this protease. Two fractions FI and FII were also unambiguous since they had no activity towards both BA*p*NA and BT*p*NA. In contrast, FIV was more likely a trypsin-like protease due to its specific hydrolysis of BA*p*NA and specific inhibition by SBTI. Two fractions FIII-1 and FIII-2 displayed much lower hydrolytic effect on BA*p*NA in comparison to FIV but were not inhibited by SBTI, thus it is still questionable if they were actually trypsin-like proteases.

On the other hand, the sequence alignment study revealed a considerable similarity between FIII-1 and FIII-2 fragments with the trypsin-like lumbrokinase fragments from *L. rubellus *and *E. fetida*. However, the sequence homology obtained in our study was expected to be higher because of the close evolutionary relationship between these earthworm species. Cho *et al*. reported extremely high conservation of the N-terminal 20-22 residues between *L. rubellus *protease fractions (Cho et al. 2004). The sequence alignment within *P. excavatus *fractions was not conducted due to insufficient information from the MS/MS data. Therefore mass spectrometric analysis for these proteases should be further elaborated to obtain their full sequences.

### The isozymes expressed strong hydrolytic activity towards both fibrinogen and fibrin

(Park et al. (2007)) discovered a protease from *Flammulina velutipes *that showed both fibrinolytic and fibrinogenolytic activity. This enzyme could perform hydrolyses without the presence of any activators, while human plasminogen is an inactive precursor and strictly requires tPA or urokinase for its conversion into fibrinolytic plasmin. In our experiment, all fractions except for FII displayed remarkable fibrinolysis, which was two to three times stronger than human plasmin (data not shown). They rapidly degraded the fibrinogen monomer as well. Therefore, the *P. excavatus *proteases would have a different catalytic mechanism towards these two substrates than human plasminogen. Moreover, each fraction experimentally displayed a distinct catalytic rate, thus probably having different kinetic parameters such as *K_M_*, *V_max_*, and *K_cat_*.

### Applicability of *P. excavatus *proteases

Pure proteins are generally less stable in water due to the absence of natural intracellular buffering and ionic conditions. Therefore, good storage conditions are required to maintain their biological activity. Nakajima *et al*. found that the protease fractions from Japanese *L. rubellus *could maintain approximately 80% of their activity after five years in 100 mM Tris-HCl buffer at pH 8 (Nakajima et al. 2000). Interestingly, our results revealed that water is more appropriate than phosphate buffer as a storage medium for *P. excavatus *proteases. In addition, the presence of plasminogen activators was declared to be unnecessary for the enzymes that could be able to hydrolyze both fibrinogen and fibrin (Park et al. 2007). These properties are favourable for convenient and cost-effective formulation of these enzymes. (Cho et al. (2004)) reported that all six protease fractions from *L. rubellus *had similar caseinolytic activity, only one of which exhibited remarkable fibrinolysis. This fraction was the first earthworm protease to be investigated for thrombosis therapy in Korea. Therefore, the proteases from *P. excavatus *characterised in the present study seem promising candidates for that purpose in Vietnam. Fraction FIII-2 is the most interesting fraction because of its strong fibrinolysis activity and high stability over long-term storage.

## Competing interests

The authors declare that they have no competing interests.
